# Radiation-Induced Lung Injury (RILI)

**DOI:** 10.3389/fonc.2019.00877

**Published:** 2019-09-06

**Authors:** Lorena Giuranno, Jonathan Ient, Dirk De Ruysscher, Marc A. Vooijs

**Affiliations:** Department of Radiotherapy, GROW School for Oncology Maastricht University Medical Centre, Maastricht, Netherlands

**Keywords:** radiotherapy, adverse effects, RILI, RILT, pneumonitis, fibrosis, lung

## Abstract

Radiation pneumonitis (RP) and radiation fibrosis (RF) are two dose-limiting toxicities of radiotherapy (RT), especially for lung, and esophageal cancer. It occurs in 5–20% of patients and limits the maximum dose that can be delivered, reducing tumor control probability (TCP) and may lead to dyspnea, lung fibrosis, and impaired quality of life. Both physical and biological factors determine the normal tissue complication probability (NTCP) by Radiotherapy. A better understanding of the pathophysiological sequence of radiation-induced lung injury (RILI) and the intrinsic, environmental and treatment-related factors may aid in the prevention, and better management of radiation-induced lung damage. In this review, we summarize our current understanding of the pathological and molecular consequences of lung exposure to ionizing radiation, and pharmaceutical interventions that may be beneficial in the prevention or curtailment of RILI, and therefore enable a more durable therapeutic tumor response.

## Development of the RILI (Molecular and Clinical Response)

**Figure 1:**
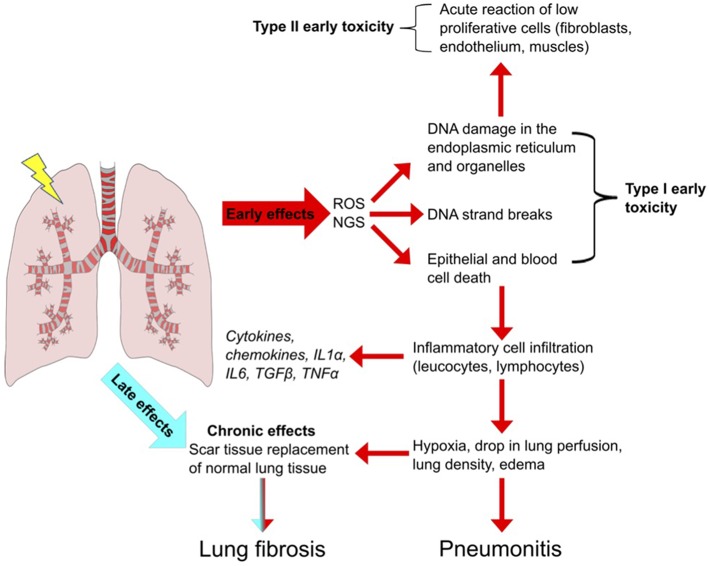
Radiation-Induced Lung Injury (RILI). Schematic overview of the important steps leading to pulmonary toxicity after radiotherapy. Radiation induces reactive oxygen and nitrogen species (ROS, NGS) which leads to DNA strand breaks and to epithelial cell death. Inflammatory cells infiltrate the affected region to remove death cells. Leucocytes and lymphocytes proliferate and produce cytokines and chemokines, leading to an inflammatory condition highly deregulated in duration and perpetuation. The persistence of the inflammatory state culminates in early reversible toxicity (pneumonitis) and can develop in to irreversible late toxicity (fibrosis).

The lung is one of the most sensitive tissues to ionizing radiation, and its susceptibility to radiation damage limits the success of radiotherapy for lung cancer treatment. The effects of lung irradiation are typically divided into early radiation toxicity, occurring within hours to a few days after RT exposure, and late radiation toxicity, occurring months to years after the treatment, which includes tissue fibrosis, necrosis, atrophy, and vascular injury [Figure 1; (1, 2)].

Two primary mechanisms cause radiation-induced tissue injury: direct DNA damage and the generation of reactive oxygen species (3). Minutes after irradiation, the damage to DNA or cytoplasmic organelles triggers intracellular signaling, leading to altered gene expression and immediate release of growth factors such as transforming growth factor ß (TGF-ß), platelet-derived growth factor (PDGF), and interleukin 1 (IL-1) (4). Additionally, ionization of water molecules generates reactive oxygen species (ROS) such as superoxide, hydrogen peroxide, hydroxyl radicals and nitrogen species (NGS) (5) that account for 60% of the total damage inflicted (6, 7). ROS can directly modify proteins and organelles but in an iron dependent manner (Fenton reaction) can produce hydroxyl radicals that cause DNA damage (8, 9). ROS also induces DNA damage in mitochondrial DNA of which cells typically have thousands of variant copies and is more sensitive to damage than nuclear DNA because lack of repair. MtDNA damage acts as DNA damage associated molecular pattern that provoke inflammation and immune responses and apoptotic cell death and is strongly associated with immune related lung diseases (10, 11) Furthermore, ROS cause cell loss, edema of the alveolar walls, increased vascular permeability and exudation of proteins into the alveolar space which further reduces the alveolar septa, and vascular integrity leading to the apoptosis of alveolar type-I pneumocytes. The human alveolar epithelium is composed of type-I and II pneumocytes, which constitute 90 and 10% of cells in the alveolus, respectively. Type-II cells are the precursors of type-I cells and synthesize and secrete the pulmonary surfactant that regulates the alveolar-surface tension. The average turnover of the lung epithelium is 4 weeks but after radiation, the sensitive type-I pneumocytes are rapidly lost, and type-II pneumocytes drive re-epithelialization of the alveolus (12). Epithelial and endothelial cell loss, due to radiation-induced cell death leads to loss of barrier function and vessel integrity thereby reducing micro-vessel density and oxygen perfusion (1, 13). These effects of ROS are counteracted by direct activation of the hypoxia-inducible factors (HIF) 1α and 2α by ROS in cells, resulting in the activation of cytokines and growth factors including VEGF that promote endothelial cell proliferation. After tumor reoxygenation, nuclear accumulation of HIFα, and enhanced translation of HIF-1-regulated transcripts occur in response to ROS. The resulting increase in HIF-1-regulated cytokine expression enhances endothelial cell radio-resistance (14).

Following apoptotic death, damage associated molecular pattern molecules (DAMPs) are released from cells triggering the recruitment of immune effector cells from the innate immune system (neutrophils, macrophages, leukocytes, lymphocytes) that infiltrate into the damaged lung, and contribute to tissue remodeling (15, 16). Neutrophils are the first to arrive to the injured site. The increased endothelial expression of intercellular adhesion molecule 1 (ICAM-1) and platelet endothelial cell adhesion molecule 1 (PECAM-1) promotes neutrophil transmigration into the damaged lung followed by lymphocytic and macrophage transmigration. The inflammatory cells produce pro-inflammatory cytokines such as interleukins (IL) IL-3, IL-6, IL-7, TNF-α, TGF-β which results in the activation of fibroblasts, leading to the initiation of additional paracrine and autocrine loops between fibroblasts, endothelia, and macrophages (17, 18). The increased concentration of macrophages enhances the production of TNF-α, stimulating IL-6 secretion, and fibroblast proliferation (19, 20). The consumption of oxygen needed for the activation of the immune cells leads to tissue hypoxia. Hypoxia promotes the generation of ROS, upregulates TGF-β, and promotes collagen formation, which reduces the elasticity of the lung alveolus (13). Furthermore, hypoxia slows down the degradation of hypoxia-inducible factors (HIF) 1α and 2α in cells, resulting in the activation of genes encoding VEGF, erythropoietin and lactate dehydrogenase 5 (21). VEGF upregulation also occurs through TGF-β stimulation via SMAD3 signaling (22), which triggers an autocrine stimulus leading to late toxicity. All these events lead to radiation pneumonitis which is an acute a reversible event in RILI. Most patients develop only radiological signs of pneumonitis without symptoms.

Density changes of the lung parenchyma are a known effect of radiation therapy that can be monitored by CT. The dose dependent density change consists of 2 phases: a transient phase peaking (3–4 months) and a fibrotic phase (after 9 months). These coincide with the time points of pneumonitis and fibrosis. Significantly, pre-treatment (23). Thus, CT monitoring of density change is an important factor when symptoms are absent. In case of symptoms such as cough and dyspnea, other causes than RILI should be excluded since up to 45% of patients have these symptoms that are not due to RILI (24). More severe cases are treated with corticosteroids with mostly fast improvement and recovery. The optimal corticosteroid schedule has never been investigated prospectively and in view of the heterogeneity of RILI, it is conceivable that a one-fits-it-all approach is suboptimal.

The mechanism of how corticosteroids suppress, or reverse radiation pneumonitis has been mostly investigated in preclinical studies. Administration of dexamethasone 4 weeks after irradiation reduces inflammatory cell infiltration and cytokine expression of TNF-α, IL-6, IL-17A, and TGF-β1 in broncho-alveolar lavage fluid improving survival (*p* = 0.0323). In the RT only group, 13 mice (65.0%) died within 180 days after RT while in the dexamethasone group only 6 mice (30.0%) died (25). Similar results were obtained with a single dose administration of dexamethasone (5 mg/kg) after 20 Gy thoracic irradiation in C3H/HeN mice which suppressed expression of pro-inflammatory cytokines, TNF-α, IL-1α, and IL-1β mRNA within 6 h after irradiation (26). Expression profiling on the lungs of 20 Gy irradiated mice, showed that CTGF (connective tissue growth factor) a central mediator of tissue remodeling, was upregulated after irradiation. Administration of FG-3019, a human monoclonal antibody that binds human and rodent CTGF, extended median survival of irradiated mice from 161 days to 300 days. Pneumonitis was reduced within 2 weeks of FG-3019 treatment and almost completely reversed by 24 weeks. FG-3019, attenuated the lung density increase after RT, improved lung function, reduced lung wall erosion, collagen deposition, and leukocyte infiltration (27). In depth analysis of gene expression changes in mouse lungs treated with and without FG-3019 and irradiation showed amelioration of RT-associated expression pattern highly enriched in macrophage, dendritic cell, mast cell, and mesenchymal transcripts. Furthermore, the administration of FG-3019, 2 days prior RT for 8 weeks, reduced RT-induced radiologic, histologic, and functional lung deficits and attenuated growth factor and matrix remodeling genes which resulted in an improved lung function and a prolonged survival (28). In patients who cannot tolerate steroids or are unresponsive, other immunosuppressive agents such as azathioprine and cyclosporine can be considered; however, evidence for their efficacy is limited to case reports (29).

TGF-β produced by inflammatory cells is the primary driver of late lung toxicity. The increase in TGF-β levels after RT accompanies elevated collagen IV gene expression (30). This collagen is associated with basement membranes of endothelial and epithelial cells, leading to septal thickening, and indicative of microvascular injury. Pneumocytes and fibroblasts also contribute to TGF-β production in response to RT (31). TGF-β exerts its pro-fibrotic role by binding the transmembrane serine/threonine kinase, TGF-β receptors. Ligand binding induces activation of SMAD a transcriptional activator of collagen (32, 33). Through the stimulation of metalloproteinase inhibitors (TIMPs), TGF-β inhibits collagen catabolism which results in collagen accumulation and conversion of fibroblasts into myofibroblasts, leading to lung architecture remodeling. The differentiation of fibroblast into myofibroblasts as a consequence of TGF-β production results in increased expression of alpha-smooth muscle actin (α-SMA) (34). Myofibroblasts may also derive from circulating bone marrow-derived progenitor cells known as fibrocytes or from epithelial cells undergoing an epithelial-mesenchymal transition (EMT) (17). In response to TGF-β, myofibroblasts secrete excess collagen, fibronectin, and proteoglycans (35), resulting in increased stiffness, and thickening of the lung parenchyma. Furthermore, the increased activity of TIMPs and decreased matrix metalloproteinase (MMP) activity (MMP2-MMP9) leads to excessive ECM deposition (36), and excess collagen. These changes lead to pulmonary fibrosis resulting in fibrotic areas susceptible to physical trauma (i.e., rupture) and gradual ischemia, which further leads to loss of respiratory capacity, tissue atrophy, and necrosis (37, 38).

## Risk Factors for RILI

The guidelines for measuring and reporting radiation toxicity in relation to dose/volume and clinical outcome have been described in the QUANTEC report: Quantitative Analysis of Normal Tissue Effects in the Clinic, that describe the development of Normal Tissue Complication Probability models (NTCP) (39). The likelihood of developing adverse lung effects after radiotherapy and the severity is strongly associated with patient characteristics and dosimetric parameters (40). Although the absolute risk of developing radiation-induced lung toxicity remains difficult to predict (41), the evaluation of the patient's clinical conditions and the potential risk factors to lung toxicity to upgrade the QUANTEC recommendations is setting the basis for a personalized treatment and setting dose-volume limits for personalized treatment [Table 1; (103)]. Radiologically abnormal Interstitial lung abnormalities are predictor for radiation pneumonitis (106). Clinical studies looking at RILI are often difficult to interpret because different endpoints are used (e.g., dyspnea score, corticoid use), and because only the maximum dyspnea is scored as an event after RT. Because approximately 50% of lung cancer patients have already some grade of dyspnea before radiotherapy, scoring dyspnea after RT, without considering baseline dyspnea, exaggerates the effect of RT on dyspnea, and proposed. Moreover, about 20% of patients have less dyspnea after RT, which is not taken into account in most models (107). Defraene et al. proposed a more accurate model for predicting radiation pneumonitis by combining dosimetric parameters with the ΔDyspnea score which is the maximum dyspnea score after 6 months corrected for baseline-dyspnea (108). Dyspnea scores are subjective, which hamper detailed quantitative analyses, including biomarker identification. The goal is the development of risk models to stratify patients according to their genetic risk for radiotherapy-induced damage and hence to more optimal personalized radiotherapy schedules (109). The single most important risk factor to develop severe radiation pneumonitis is interstitial lung disease (ILD) which can be quantified by the uptake of FDG in the lungs. In a retrospective study of 101 NSCLC patients a CT (4D-CT) scan directly followed by an [18F]FDG-PET scan was performed before radiotherapy treatment. Patients with high [18F]FDG uptake in 5 to 10% of the lungs before RT were more likely to develop radiation toxicity than patients with a low uptake. Furthermore, in patients with RILI the [18F]FDG uptake was higher in the lower lobe of the lung than in other regions. These results suggest that identifying patients at high risk for RILI on the basis of a pretreatment [18F]FDG-PET-CT can be used to individualize treatment (106, 110).

**Table 1:** Dosimetric and biological parameters in radiation-induced lung toxicity.

	**Parameters**	**Risk increase**	**References**
Patients characteristics	Age	over 65	(41–46)
	Gender	female	(44, 47, 48)
	Smoking	non-smokers	(43, 48–53)
	Pre-existing lung diseases	ECOG performance 3–4	(45, 46, 54–62)
	Genetic predisposition	SNPs in various genes	(63–74)
	Tumor location	Base, the upper half of the lung, the region adjacent to the pleura	(51, 70, 75–79)
	Low KPS	Radiation pneumonitis	(41, 48, 77, 78)
Dosimetric parameters	Chemotherapy	Most chemotherapies	(41, 46, 48, 56, 61, 79–90)
	Chemo-XRT schedule:	Sequential > concurrent fraction size >2.67 Gy	(46, 48, 61, 83, 91)
	Targeted therapies	TKI monotherapy and with RT	(92–96)
	Mean Lung Dose (MLD)	Higher MLD	(97–103)
	Dose to the heart	Undetermined	(104, 105)

*Patient's characteristics (age, gender, smoking status, pulmonary status, genetic predisposition) and dosimetric parameters (chemotherapy, radiotherapy, tumor location, lung volume, NTCP, MLD) affect the probability of radiation-induced lung toxicity. >, major; NTCP, normal tissue complication probability; MLD, mean lung dose*.

### Patient's Characteristics

In lung cancer patients, the incidence of adverse effects after radiotherapy is related to clinical factors and a variety of dosimetric parameters (111). Age is one of the main factors associated with radiation-induced lung toxicity (45). Older patients (>65 years old) have less tolerance to RT and a major risk of developing adverse effects (41, 77). A multivariate analysis of 369 patients with an age > 65 years, with stage III non-small cell lung cancer (NSCLC), revealed how age influence both grade 2 (OR = 1.99), and grade 3 radiation pneumonitis (OR = 8.90) (41). Similar results were confirmed by others, in a prospective study with 96 patients who received three-dimensional conformal radiotherapy (3D-CRT) for stage IA to IIIB NSCLC (44). Furthermore, grade 4 + toxicity occurred in 62% of NSCLC patients <70 years compared with 81% of elderly patients (*P* = 0.007) and Grade 4 + toxicity occurred in 1% of those younger than 70 years, compared with 6% of those older than 70 (*P* = 0.02) (112). This is primarily due to the fact that older patients have more comorbidities that are a risk factor for RP, than younger patients (46). Despite age being a strong risk factor, a threshold value has not been determined due to additional risk factors such as smoking status, and pulmonary function.

The effect of gender on RILI is still unclear (77). Women have smaller lung volumes and more often develop an autoimmune disease which increases their risk of RILI. A univariate analysis of 148 lung cancer patients with good performance status (ECOG 0–1) treated with chemo-radiation confirmed that the risk of severe pneumonitis was significantly higher in women vs. men (15% in women vs. 4% in men) (48). Among 214 consecutive patients with locally advanced NSCLC that received 3D CRT, gender was a predictor for the grade ≥2 group only (OR = 0.32, *p* = 0.028) (47). Other studies do not show a significant association between gender and RP risk, probably due to the contribution of different factors such as pre-existing diseases, or radiation schedule (46, 70, 113). Further studies are needed to clarify the role of gender as a clinical risk factor for RILI.

### Pre-existing Disease

In contrast to the causative role of smoking and lung cancer, tobacco use seems to have a protective role against pneumonitis in lung cancer patients treated with chemo-radiation (50, 51). The frequency of Grade 3+ pneumonitis was higher in long time quitters in comparison to recent quitters or current smokers (72). In stage I–IV lung cancer patients (*n* = 182) treated with radical (chemo) radiotherapy the dyspnea grade was higher in patients who quit smoking compared to active smokers. A possible explanation may be that ex-smokers quit smoking because of the appearance of dyspnea. However, for a correct interpretation of the results the baseline dyspnea score has to be taken in to account in order to avoid false positive results. In 576 patients with stage III, NSCLC treated with radiotherapy the incidence of radiation-induced pneumonitis was higher (grade ≥3 pneumonitis) in non-smokers (37% at 1 year) in comparison to smokers (14% at 1 year). Patients who quit smoking before diagnosis showed an intermediate incidence of RP (23% at 1 year) although dosimetric parameters were not taken into account (52). The long-term exposure of the lung to chemicals in tobacco smoke destroys the lung tissue due to the replacement of the elastic walls with a fibrotic structure and the attraction of pro-inflammatory mediators. On the other hand, smoking leads to immunosuppression causing reduced antibody response against carcinogens compared to non-smokers (53, 114). The protective role of smoking is therefore probably related to reduced sensitivity of the damaged lung compared to a non-smokers healthy lung (48, 49, 53). Furthermore, a carbon monoxide–induced hypoxia caused by tobacco results in less ROS generation after radiolysis and therefore less lung DNA and tissue damage (53).

Significant differences in radio-sensitivity are associated with single nucleotide polymorphisms (SNP) that affect cell survival, DNA damage response, and DNA repair genes (71). SNPs in ATM, IL-1A, IL-8, TNF, TNFRSF1B, MIF rs2868371, rs1800469, TGF-β, TNF-α, VEGF, XRCC1, APEX1, IL-6 are associated with a 2.16-fold increased risk of lung injury upon irradiation (72). SNPs in IL-13 with 2 variant alleles rs20541 or rs180925 were approximately 3-times more likely to develop pneumonitis compared to those with wild-type genotypes. SNP rs10711, located in the 3′UTR region of CDK1 (encoding for cyclin-dependent kinase 1) was significantly associated with a higher risk of pneumonitis (OR = 2.67, 95% CI = 1.26–5.63, *P* = 0.010) (115). SNPs, in IL-1A, IL-8, TNFRSF1B, MIF, and NOS3 are also associated with 3.16-fold increased risk of radiation pneumonitis (72). Including SNPs in RP risk models, improves the discrimination accuracy especially when including SNPs with a high allele frequency or larger effect size. Therefore, stratification based on individual genetic differences in NTCP models could be an interesting approach to select patients at lowest risk for radiotherapy complications (74). However, despite numerous studies (SNP) associations have rarely been reproduced in independent validation studies. For example initial studies reported strong associations between TGF-β SNP and radiation pneumonitis but could not be validated in independent cohorts (55, 116, 117). Development of robust, standardized and quantitative endpoints integrating GWAS and gene-expression are needed before radiogenomics will be useful to predict radiation sensitivity (118).

## Treatment Related Risk factors

### Lung Function

Lung function is evaluated by assessing different parameters [forced expiratory volume (FEV), forced vital capacity (FVC), diffusing capacity of the lungs for carbon monoxide, total lung capacity (TLC), lung volume (LV)], and how they change before and after radiation. A decreased value of these parameters is an indicator of decreased pulmonary function and of capillary alveolar changes (DLCO), bronchial obstruction (FEV) (55), and lung stiffening (FVC, TLC) (45, 54, 57). Pre-existing lung disease strongly increases the risk for radiation pneumonitis after thoracic radiotherapy (58, 59, 106) by exacerbating inflammation and destruction of the connective tissue scaffold (60). Most lung cancer patients develop COPD, emphysema and therefore have poor pulmonary function (ECOG 3–4). In a multivariate analysis, Rancati et al. showed that lung cancer patients suffering from COPD receiving fractionated radiotherapy had 24.1% more lung toxicity compared to patients with no COPD (61). Similarly Kimura et al. found a strong correlation between the grade of pulmonary emphysema (PE) (grades 0–3) and radiation pneumonitis, ranging from 16.5% (grade 0) to 54.0% (grade 3) (62).

### Dose

Dosimetric parameters (irradiated volume, mean lung dose (MLD), dose delivered, schedule, tumor location) are risk factors for RILI (119). Irradiating larger volumes of the lung causes compromises lung function (79, 113). Improvements in three-dimensional treatment planning systems describe the relationship between irradiated lung volume and the probability of tissue complications. Robnett et al. demonstrated that in a group of 540 patients, the risk for grade 2 pneumonitis strongly correlated with MLD (48). Other studies underline how V20 and V30 (lung volume receiving 20 or 30 Gy, respectively) are the only parameters significant in predicting RP (79, 113). One significant limitation is that most studies determine the DVH (Dose Volume Histogram) but do not consider lung movement and deformation resulting from breathing (43). Hernando and Guerrero et al. found that an MLD < 10 Gy is associated with a 10% radiation pneumonitis rate but this increases to 16% with an MLD of 11–20 Gy (43, 101). The NTCP model describes the probability of RP as a function of a sigmoidal dose relationship (39). To calculate the likelihood of lung injury this approach relates the tolerance dose for lung irradiation and the slope of the dose-response curve (120, 121). In a study of 42 lung cancer patients treated with fractionated radiotherapy, with a dose of 67 Gy, the NTCP average values were 73% for the patients with RP, and 25% for those without lung comorbidities (102). Thus, NTCP could be used for the optimization of the treatment plan (122).

While the mean dose to the lung is a key risk factor for RILI, the dose to the heart also influences pulmonary toxicity. Using high energy proton irradiation van Luijk et al. demonstrated that co-irradiation of the heart and lung significantly increased breathing rate as surrogate marker for lung function in rats compared to lung irradiation alone (123). Further work in preclinical models from the same group showed that that lung and heart irradiation through different mechanisms enhance lung toxicity by increasing hypertension and inducing vascular pulmonary damage and that both need to be avoided that to reduce lung toxicity after irradiation (124). Indeed using precision irradiation of the heart limiting dose- to small volumes in the lung induced lung pneumonitis in a dose-dependent manner in the absence of heart toxicity in mouse models (125). Also in patients reduced cardio-pulmonary function further exacerbates radiation induced lung toxicity (104). In large cohort studies no significant association between heart dose and RP was found (105). Quantitative parameters independent of dyspnea and cough that may result from pre-existing lung disease rather than a consequence of cardiac damage are needed.

### Tumor Location

Tumor location is one of the main predictors for RP development in preclinical and clinical studies. In C3Hf/KAM mice where 70% of the lung was irradiated with a fixed dose (22 Gy), irradiation of the midlung was associated with less morbidity from RP than radiation of the base, the upper half of the lung, and the region adjacent to the pleura (75). In a retrospective study of 60 lung cancer patients that received chemo-radiation, RP was more frequent after irradiation of the base of the lobe (70%) rather than after irradiation of the upper lung lobes (20%) (51). In the study of Seppenwolde et al. the risk of RP has been evaluated in relation to the regional dose distribution in 106 lung cancer patients that received fractionated radiotherapy (2 Gy/fraction) was determined. Dividing the lung into different sub-volumes the incidence of RP was higher in posterior central and peripheral regions in comparison to anterior and contralateral zones (76). Another study of 324 lung cancer patients confirms the higher incidence of lung injury following the exposure of the lower region compared to the upper one, suggesting the importance of superior-to-inferior tumor position as a significant variable (70). Possible explanations for the different regional radio-sensitivity might be related to the better oxygenation, perfusion, and ventilation of the lower pulmonary region (77).

### Systemic Treatment

There are conflicting results on the effects of concurrent, concomitant, or neoadjuvant chemotherapy on radiation toxicity. Neoadjuvant chemotherapy shrinks the tumor volume before RT and reduces the PTV (planned treatment volume), and the risk of RILI. A dosimetric analysis of 23 patients with stage IV small cell lung cancer receiving platinum-based chemotherapy as neoadjuvant showed that 30 had a 20% reduction in tumor volume after induction chemotherapy. This translated into a 5% reduction in risk for RILI (85), suggesting that neoadjuvant chemotherapy can reduce RILI (19, 79). In contrast, a retrospective study of 223 patients treated with concurrent chemoradiotherapy did not show a significant effect on RP (<3) among treatments (with and without RT) (126). The difference might be explained by different patient selection criteria, radiotherapy dose, chemotherapy doses, and clinical schedule (concurrent vs. neoadjuvant). Paclitaxel, taxol, dactinomycin, cyclophosphamide, doxorubicin, mitomycin C, gemcitabine, and irinotecan have all been reported to increase the risk of pulmonary toxicity when combined with fractionated radiation (56). In a phase I/II study of 24 patients with NSCLC treated with paclitaxel/cisplatin concurrent with radiotherapy, the incidence of pulmonary toxicity was higher in patients treated with chemoradiation compared to patients receiving both monotherapies (80). In a retrospective study of 84 lung cancer patients who received 3D-CRT, grade >2 RP was 3-fold higher in patients with mitomycin (31.2%) than those with RT only (61).

In NSCLC patients, radiation pneumonitis was a dose-limiting toxicity when gemcitabine was administered at a dose of 50 mg/m2 twice-weekly with concurrent radiotherapy (81) and the incidence of Grade >2 RP was several fold higher among patients who received irinotecan (56%) compared with those who did not (14%) (56, 82). RILI is highly dependent on the treatment schedule. Sequential chemotherapy is associated with an increased risk for RP compared with a concomitant regimen (46, 83). Fraction size is another critical parameter in RILI. Fractions >2.67 Gy enhance the risk of RILI compared to lower daily fractions (84).

### Targeted Therapies

Tyrosine Kinase inhibitors (erlotinib and gefitinib) against EGFR are first-line treatment for patient with EGFR-mutated non-small cell lung cancer. A large meta-analysis with more than 15 thousand patients showed it increases the incidence of pneumonitis with ethnic differences in susceptibility (127). No differences were found in efficacy or toxicity between Erlotinib and Gefitinib in randomized phase trials of EGFR mutated NSCLC (95). Third generation TKI's such as Osimertinib are more potent than Erlotinib and Gefitinib and increase progression free survival and overall survival with comparable overall toxicity, although interstitial lung disease, and cough symptoms were elevated in the Osimertinib group (96). Acute side effects from TKI can be managed by cessation of treatment or with corticosteroids (128). There are a limited number of studies with low number of patiens and case reports describing increases in interstitial lung disease and pneumonitis with concurrent thoracic radiotherapy and erlotinib in the curative (73, 93), and palliative setting (94). Larger studies are needed to understand the understand the full scope of these adverse effects.

### Immunotherapy

Recently, by combining immune checkpoint inhibitors with chemoradiotherapy significant responses and prolonged survival are seen in treatment-refractory advanced Stage III NSCLC (129). There is mounting evidence that radiation may induce expression of checkpoint receptors on normal tissues as well (130, 131). NSCLC cancer patients often have pre-existing conditions such as COPD, asthma, and emphysema that are associated with inflammation and hypoxia that may upregulate PDL-1 in normal tissue resident cells or in infiltrating immune cells (132). Autoimmune-related response are strong predictors for outcome in patients treated with checkpoint inhibitors (133). However, they are also associated with treatment related side-effects. In a meta-analysis of serious adverse events of PD-1 and PD-L1 inhibitors in clinical trials, pneumonitis was among the most frequent common causes of death (134, 135). Stereotactic high dose radiotherapy is standard of care in inoperable node-negative NSCLC with manageable lung toxicity (136). Hypofractionated radiation has been shown to convert “cold' tumors to” hot 'inflammatory tumors by activating damage associated pathways and releasing pro-inflammatory factors that boost an anti-tumor T-cell response (137, 138). Indeed, pre-treatment immunological status predicts response to stereotactic hypofractionated RT in patients (139). With the promise of combining immunotherapy and radiotherapy we may see an increase of stereotactic radiation in the thorax. Thus, it will be important to monitor for increases in immune-related side effect as well. To date, immune-checkpoint inhibitors show objective responses in up to 30% of patients.

## Intervention and Prevention

Approaches are sought to prevent, dampen, or delay late normal tissue complication. Conformal radiotherapy, as well as intensity-modulated radiotherapy (IMRT) and particle (e.g., carbon, proton) therapy, are used to improve irradiation of tumor volumes while sparing the healthy tissue. While these approaches substantially contribute to more precise tumor irradiation dose, exposure of the healthy tissue is inevitable. The study of a cohort of 188 NSCLC patients that underwent (chemo-)radiotherapy with IMRT or VMAT show grade four and grade five late toxicity only in VMAT (Volumetric Arc Therapy) treated patients when compared to IMRT. Grade >3 lung pulmonary toxicity nearly doubled in the VMAT group compared to the IMRT treated patients. The differences may be related to the different dose distribution characteristics which may result in differences in radiation exposure (140). In the study of Jegadesh et al. IMRT was associated with an improvement in median overall survival and 5-year survival rate (17.2 vs. 14.6 months; 19.9% vs. 13.4%, *p* = 0.021). Different studies demonstrate the ability of IMRT to reduce mean lung dose, lung V20, maximum dos to the spinal cord and multiple heart dosimetric parameters and improves quality of life, dosimetry, and toxicity in comparison to 3D CRT in the treatment of locally advanced NSCLC (141). Furthermore, IMRT may improve dosimetric parameters via increased dose conformity to the target volume. A more detailed description of technological advances in precision irradiation falls outside of the scope of this review, and the reader is referred to other studies (142, 143).

Biological approaches exploit differences in radiation- sensitivity between tumor and healthy tissue. These include the use of radiation protectors, modifiers, or mitigators. These drugs are specific for acute or late responding normal tissues, ideally without any protective effect on the tumor cells. There are three types of pharmaceuticals that can be administered at different stages of treatment. Firstly, radio-protectors are given before radiation exposure; secondly, radio-mitigators are given during or immediately after radiotherapy but before the appearance of toxicity; and thirdly treatments can be administered after the appearance of toxicity to attenuate progression or reverse the damage (144, 145). However, multiple approaches are often used to obtain successful results, due to different factors that contribute to RP (lung sensitivity, cellular turnover, dosimetric, and clinical parameters) (40).

### Radioprotectors

Amifostine is a thiol prodrug whose damage repair mechanism acts through a hydrogen atom donation and to date, the only thiol-drug approved for clinical use (146). It is administered as an inactive prodrug that after de-phosphorylation by Alkaline Phosphatase is activated (147). It is relatively specific for normal cells due to the relatively high expression of Alkaline Phosphatases in normal tissue vs. tumor cells. Furthermore, the low pH of the tumor microenvironment inactivates amifostine contributing to its normal tissue specificity (148). The thiol group of amifostine plays a key role in activating redox-sensitive transcription nuclear factor kappa-light-chain-enhancer of activated B cells (NFκB), resulting in an increased expression of Superoxide Dismutase 2 (SOD2) (149). Increased SOD enzyme expression further contributes to ROS neutralization into less reactive products (hydrogen peroxide and oxygen) (149). Once oxidized amifostine induces oxygen depletion, by increasing oxygen consumption in normal cells making them more resistant to permanent radiation-induced DNA damage. Antonodau et al. reported a significant reduction in esophagitis > grade 2 and RP (grade 3) (*P* <0.001) in lung cancer patients receiving thoracic radiation post amifostine (340 mg/m2 IV before daily RT) (150). Hildebrandt et al. showed that both esophagitis > grade 3 (16 vs. 35%), and radiation pneumonitis (0 vs. 16%; *P* < 0.02) was significantly lower in 62 patients receiving neoadjuvant amifostine, concurrent chemotherapy (cisplatin and oral etoposide), and hypofractionated radiotherapy (1.2 Gy bid to 69.6 Gy) (72). Although amifostine is generally well-tolerated, side-effects include nausea, fever, hypotension and allergic reactions in a dose-dependent manner. The treatment is rarely interrupted however, suggesting a safe and tolerable profile (148, 151).

ROS can also be neutralized by engineered nanoparticles which have shown radioprotective effects in preclinical models. For example, the administration of Manganese Superoxide Dismutase-Plasmid Liposomes (MnSOD-PL) in a single dose, before RT has been shown to decrease the magnitude and duration of cytokine production, and thiol and lipid peroxidation *in vivo* studies. Intratracheal administration of MnSOD-PL in C57Bl/6 mice with orthotopic Lewis Lung carcinoma 24 h before whole thorax irradiation (18Gy) resulted in tumor radiosensitization but spared the surrounding normal tissue, prolonging mice survival (152). Similar results were shown by Carpenter et al. who showed that inhalation of MnSOD-PL 24 h before radiotherapy increased survival in 20 Gy irradiated mice compared to controls. Furthermore, a decrease in alveolitis, lung fibrosis, and weight loss was observed (153). Together with SOD nanoparticles, porphyrins also showed a reduction in radiation damage. Vujaskovic et al. showed a reduction of RT lung injury after the supplementation of AEOL 10113 [manganese (III) mesotetrakis (N-ethylpyridinium-2-yl) porphyrin (MnTE-2-PyP(5+)] in 28 Gy irradiated rats. AEOL 10113 significantly reduces the severity of RT-induced lung injury by reducing collagen deposition, pro-fibrogenic cytokines and TGF-β in irradiated rats (154). Inactivation of ROS can be accomplished by administration of cerium oxide nanoparticles (CNPs) (155). Intraperitoneal injection of high dose (10 μM) CNP-18, and CNP-ME, 2 h post irradiation for 4 weeks improves 15 Gy whole-thorax irradiated CBA/J mice survival (survival rates of 40%) compared to irradiation alone (10%). Additionally, a reduction of vascular damage, collagen deposition, and inflammatory response was recorded (156).

Genistein is a soy-derivative and isoflavone, which acts as radical scavenger and protein kinase inhibitor (157). In irradiated murine lungs, it exerts anti-inflammatory and antioxidant properties by limiting macrophages infiltration, reducing micronuclei formation, lowering ROS levels, and preventing DNA damage (158). Genistein supplementation before and immediately after radiotherapy protects from the onset of pneumonitis and reduces fibrosis in C57Bl/6 mice (159). Humanetics Corporation is developing BIO300SOD/ a nanoparticle formulation of Genistein to prevent pneumonitis and fibrogenesis. The efficacy and the toxicological profile of BIO300 has been tested *in vivo* models while its pharmacokinetic and safety has been evaluated in a phase I safety and pharmacokinetic trial (NCT 00504335).

Targeting DNA damage caused by ROS is another promising strategy to reduce radiation side effects. Intraperitoneal administration of the catalase mimetic Eukarion (2–30 mg/kg) post irradiation (10 to 20 Gy ^60^Co γ-rays) reduced micronuclei induction, when given after irradiation (160). Berberine and other ROS scavenger at 20 mg/kg once a day for 6 weeks reduces the incidence of lung injury in NSCLC patients treated with 2 Gy to a total of 60–70 Gy. Improved pulmonary function and decreased intercellular adhesion molecule 1 (ICAM-1), and TGF-β levels were observed (161). Nitroxides are potent free radical scavengers and Tempol (4-hydroxy-2,2,6,6-tetramethylpiperidine-1-oxyl), has shown a protective role against ROS in *in vivo* models (148). Preclinical studies showed a radioprotective effect of systemic administration of Tempol (275 mg/kg) in C3H mice exposed to whole body irradiation without any effect on tumor growth (162). The paramagnetic property of the oxidized Tempol permits non-invasive Magnetic Resonance Imaging (MRI) resulting in image contrast enhancement (40). Thus, the concentration of its radioprotective state and site of accumulation in tissue provides essential information about the compound and the radiation treatment.

Pentoxifylline is a xanthine derivative agent which down-regulates the production of pro-inflammatory cytokines, particularly TNF-α, and inhibits platelet aggregation. Pentoxifylline (400 mg/3 times daily), in patients with lung and breast cancer receiving a daily dose of 2 Gy, 5 days/week, protects against both early and late lung toxicity (163). In preclinical studies administration of pentoxifylline (500 mg/l), 1 week before irradiation in C57Bl/6 mice receiving whole thorax irradiation (12 Gy) reduces the RP phase (164). The radioprotective effects are related to the increase in locoregional blood flow. Pentoxifylline inhibits cAMP phosphodiesterase causing a cascade of events reducing leukocyte adherence to endothelial cells, minimized platelet aggregation and dilatation of capillaries through enhanced prostacyclin synthesis. Consequently, blood viscosity, and systemic vascular resistance are reduced (165). High levels of cAMP reduce the release of bioactive TNF-α by downregulating the expression of the TNF-α gene.

### Radiomitigators

Targeting inflammation represents an alternative approach to reduce adverse effects during radiotherapy. In 18 Gy irradiated C57Bl/6 mice, intraperitoneal injection of methyl prednisone, an immune suppressor (40 mg/kg a day), for 7 days reduces TGF-β1 and TNF-α in lung tissue at 9 weeks after radiation exposure delaying the development of fibrosis at 12 weeks after irradiation (166). Statins (HMG-co-reductase inhibitors) ameliorate endothelial function by increasing the concentration of endothelial nitric oxide synthase (eNOS), which promotes anti-inflammatory signaling, prevents apoptosis, increases vasodilatation, and reduces platelet adhesion (157). Treatment with Ulinastatin for 3 days pre- and 4 days post-irradiation in 20 Gy irradiated Sprague Dawley rats attenuated pulmonary injury compared to the control group which developed severe fibrosis (167). Although the mechanism of action of Ulinastatin is not fully understood, it caused the downregulation of pro-inflammatory chemokines and alleviated pulmonary edema and inflammation of the alveoli (168). Lovastatin shows similar results. Statins have also been shown to reduce O_2_ consumption, which could counteract the protective effect of statins by reducing inflammation. In C57Bl/6 mice exposed to a single dose, 15 Gy whole thorax irradiation Lovastatin reduced radiation-induced pneumonitis by exerting anti-inflammatory, anti-apoptotic and antifibrotic effects (169). A recent study shows how Ethyl Pyruvate (EP), an effective inflammatory injury ameliorator, alleviates radiation injury in C57Bl/6 mice exposed to whole lung irradiation (16 Gy). Intraperitoneal injection of EP once every day for 1 month after RT was associated with a lower histological grade of inflammation and reduced pro-inflammatory cytokines, IL-1β, IL-6, and GM-CSF in irradiated mice. Furthermore, EP suppresses the production of TGF-β1 showing anti-fibrotic effects (54).

ACE (angiotensin-converting enzyme) inhibitors and angiotensin-2 antagonists, normally used to regulate blood pressure and prevent cardiovascular diseases, have been shown to diminish radiation-induced tissue damage in preclinical models. Robbins reviewed the use of the renin-angiotensin-system agents (RAS agents) for mitigating late radiation effects (144, 170). Angiotensin II Receptor Antagonist (AT_2_RA 158, 809) and the ACE inhibitors (Captopril and Enalapril) attenuate the effects of radiation damage by targeting the oxidant, inflammatory and fibrogenic pathways. Angiotensin II regulates TGF-β and α-smooth muscle actin (SMA), two proteins with a critical role in the pathogenesis of pulmonary fibrosis (171, 172). Enalapril reduces vascular remodeling and decreases levels of TGF-β (173). Supplementing RAS agents (ACEIs Captopril, CL 24817, Enalapril, and CGS 13945) in irradiated Sprague Dawley rats has been shown to reduce expression of endothelial dysfunction markers (chemokine secretion and leukocyte adherence, cell permeability, enhanced low-density lipoprotein oxidation, platelet activation). The AT_2_RA 158,809, Captopril and Enalapril, significantly delay radiation-induced lung injury. The incidence of Grade > 2 pneumonitis was significantly lower in 62 patients with stage I or III treated with ACEIs during thoracic irradiation compared to 100 non-users (2 vs. 11%) (174).

Curcumin (diferuloylmethane), a natural compound extracted from Curcuma longa, inhibits NSCLC metastasis by blocking the Adiponectin/NF-κB/MMPs Signaling Pathway (175), and reducing cell viability, invasion, and migration. This was shown in the metastatic 95D NSCLC cell line (176). Curcumin attenuates radiation-induced lung inflammation and fibrosis in 18 Gy irradiated rats by exerting anti-inflammatory effects, reducing macrophage infiltration and attenuating the increase of alveolar septal thickness after radiation. Furthermore, collagen accumulation, TGF-β content, and CTGF and NF-κB expression levels were significant reduced in curcumin treated rats (177).

Several growth factors act as radiation damage mitigators when supplemented close to the time of irradiation. Among these compounds Keratinocyte Growth Factor (KGF) is the most common. KGF is a paracrine growth factor which stimulates the proliferation and differentiation of alveolar type 2 cell and protects the lung from radiation injury. Liu et al. showed, in Sprague–Dawley rat models (20 Gy), how radiation-induced fibrosis is delayed after KGF administration, stimulating cell proliferation, inhibiting inflammatory response and decreasing lipid peroxidation as a direct result of ROS. KGF also promotes alveolar fluid clearance, decreases lung edema after pulmonary damage and reduces the reactive oxygen species (161). KGF protects against increases in endothelium permeability, decreasing lung edema driven by hydrogen peroxide in human airway epithelial cells (178). Despite its safety profile and efficacy in treating RILI, its instability, cost and inability to enter the distal lung have limited its use. Better results are reported with Palifermin a recombinant human KGF which showed promising results in clinical trials where it decreased the incidence and duration of dysphagia in patients with an unresectable stage III NSCLC treated with concurrent CT/RT (179).

### Cell Based Therapies

Stem cell therapy has the potential to repair and restore tissue function from the adverse effects of radiotherapy in multiple tissues (180). However, there are limited preclinical studies and no clinical experience yet that guide the optimal timing of transplantation of stem cells and application to early or late radiation toxicity in lung.

Bone marrow derived mesenchymal stem cells (MSCs) have demonstrated great promise in regenerative medicine including in the lung. Due to their anti-inflammatory properties and enhanced repair capacity, MSCs are used for the treatment of inflammatory diseases and COPD, resulting in improved lung architecture, decreased apoptosis and increased cell proliferation and could be a promising therapeutic approach to mitigate radiation-induced pneumonitis. MSCs have been shown to successfully migrate toward the injury site in the lung after irradiation and adopt lung cell phenotypes (181, 182). In C57Bl/6 at 25 weeks after 15 Gy whole thorax irradiation, MSC infusion at 24 h and 14 days after RT protected the irradiated lung from severe radiation-induced vascular endothelial cell (EC) damage and delayed EC loss. Radiation-induced increase in infiltration of myeloid cells was also significantly reduced in MSC-treated animals at 25 weeks after whole thorax irradiation, which might be due to the protection of lung EC. Furthermore, MSC-derived cell culture efficiently rescued cultured lung EC from the radiation-induced side effects in short-term and long-term survival assays, indicating the protective contribution of MSC-secreted factors. Administration of MSCs after RT restores SOD1 levels in irradiated murine lung tissue, likely contributing to their protective effect. Improved vascular function and normalization of immune cell infiltration, favors both prevention and recovery from radiation injury to vascular and other resident lung cells. However, further studies are required to better understand the effects of MSCs on bronchial-alveolar and epithelial cells in order to develop MSC based therapeutic strategies (183). Adipose-derived mesenchymal stromal cells (Ad-MSCs) also have significant potential for clinical application. The delivery of Ad-MSC through the tail veins 2 h after irradiation (15 Gy) in Sprague-Dawley rats attenuates radiation-induced lung toxicity by blocking inflammatory, apoptotic, and fibrotic responses. Ad-MSc were associated with decreased serum IL-1, IL-6 and TNF-α expression within the first 28 days after irradiation and decreased fibrotic markers (TGF-β, CTGF, α-SMA, and Col1a1). Hydroxyproline content was a direct index reflecting lung fibrosis, suggesting that stem cell therapy delayed fibrosis. Thus, Ad-MSCs have therapeutic potential in RILI management as well (184).

Induced pluripotent stem cells (iPS) are a useful tool in regenerative medicine (185). The pathways that play a leading role during lung embryogenesis and morphogenesis may also be involved in the regeneration of lung tissue after injury (186). The murine lung developmental process has been elucidated through technologically advanced techniques such as whole transcriptome analysis (187, 188). Recapitulating the sequence of the developmental stages during lung development represents a promising approach to differentiate pluripotent stem cells into lung lineages. The differentiation of induced pluripotent stem cells into endodermal progenitors occurs throughout the stimulation of the Nodal signaling pathway (189). Consequently, the dual inhibition of TGF-β and BMP4 pathways and the supplementation of WNT, RA, and BMP leads to NKX2+progenitors, which further differentiate toward bronchial (Sox2^+^) and alveolar (Sox9^+^) lung lineages (185, 190). This process is known as “directed differentiation” and culminates with NKX2+ progenitors, from which all lung epithelia derived. These derived progenitors can re-populate decellularized whole lung scaffolds (186). Furthermore, the generation of ESC from patients with cystic fibrosis allows their use for lung pathologies (191) although their clinical use has not yet been documented. An understanding of the iPS mechanism of action and the molecular mechanisms of lung repair may promote their use in the irradiated lung to facilitate stem cells spreading over the damaged tissue.

Although several cell biology studies are based on submerged culture procedure, they are far from mimicking the *in vivo* lung microenvironment. 3D models such as lung organoids are a useful tool to answer several questions about lung regeneration. Broncosphere culture assays have been performed using both iPS and lung basal cells (192). Fluorescence-activated cell sorting allows the isolation of P63, NGFR, K5 positive stem, and progenitor lung epithelial cells that can be cultured in Matrigel which provides a three-dimensional structure to study the mechanisms involved in basal cell renewal. The same model can be used to investigate the mechanism of lung regeneration after irradiation in an experimental condition closer to reality (193). Three-dimensional scaffolds have been generated from synthetic or biomimetic materials to develop *ex vivo* lung parenchyma and vascular system and could be exploited for transplantation to produce functioning lung tissue in animal models (194).

### Translation From Preclinical Models

With the diversity of preclinical models and differential sensitivity, breadth and penetrance of radiation-induced lung toxicity, there is a need for a consensus on preclinical models that best resemble aspects of human disease phenotypes (195). Most models use single dose irradiation rather than clinically relevant fractionated irradiation, and there are significant strain-differences in the sensitivity to pneumonitis and fibrosis (196, 197). Treatment toxicity should also be measured in immune-tolerant orthotopic tumor bearing models using clinically relevant combination-treatment schedules and (surrogate) endpoints. Furthermore, the dose to other organs at risk in the irradiation field such as the heart influence pulmonary function and indirectly contribute to adverse effects and should be appropriately modeled as well (198). In this respect dose calculations on human tissues irradiation studies with MeV beams cannot be directly translated to mouse preclinical irradiation studies using KeV beams (199). The use of preclinical micro-irradiators with integrated CT imaging are a new tool to evaluate radiation-induced toxicity closer to clinical irradiation set-up. These small animal iGRT systems enable precision irradiation to lung subvolumes and demonstrate that CT density correlates with histopathological factors of lung remodeling (200, 201) although this may not be sensitive enough to measure small reductions in lung remodeling when using anti-fibrotics (202). These studies are important in parallel to the development of treatment planning and delivery for orthotopic lung tumor models (203) Standardization of these preclinical irradiation platforms is crucial and guidelines have recently been proposed (204).

## Concluding Remarks

Modern precision radiotherapy techniques may reduce normal tissue exposure to radiation, but many patients still experience adverse effects. Radiation-induced toxicity is dependent on many parameters, such as the tissue location, the functional status of an organ, the dose and irradiated volume and other factors. In most cancers, radiotherapy is combined with systemic treatment concurrently or concomitantly, and in many cases, they sensitize to radiation-induced lung injury or worsen pre-existing co-morbidities. There is a high need for biomarkers that can predict responders and select those patients that will only suffer from side effects. In patients individual radiation sensitivity is a quantitative and tissue-specific trait and large patient numbers and standardized outcome measures are essential as well as independent validation cohorts to identify QT-loci and the inclusion of ‘real world' data (205). While such studies provide hypothesis for future mechanistic investigation it is not yet evident how this can be translated into radiotherapy practice. One potential application is that such ‘sensitive' signatures may be used for patient selection to receive more precise but also more expensive proton therapy while keeping the maximum dose to the tumor (206). More research is therefore needed to understand which preclinical models should be exploited to identify crucial parameters that contribute to radiation toxicity and how these can be used to inform the design of clinical studies were radiation therapy is combined with other treatments to minimize radiotherapy induced toxicity at optimal tumor control.

## Author Contributions

LG, DD, and MV wrote the manuscript. LG and JI prepared the figures and tables. MV supervised the work.

### Conflict of Interest Statement

The authors declare that the research was conducted in the absence of any commercial or financial relationships that could be construed as a potential conflict of interest.
